# Autoimmune Polyendocrinopathy Candidiasis Ectodermal Dystrophy: Insights into Genotype-Phenotype Correlation

**DOI:** 10.1155/2012/353250

**Published:** 2012-10-22

**Authors:** Donatella Capalbo, Lucia De Martino, Giuliana Giardino, Raffaella Di Mase, Iolanda Di Donato, Giancarlo Parenti, Pietro Vajro, Claudio Pignata, Mariacarolina Salerno

**Affiliations:** ^1^Department of Pediatrics, University of Naples Federico II, 80131 Naples, Italy; ^2^Department of Pediatrics, University of Salerno, 84081 Salerno, Italy

## Abstract

Autoimmune polyendocrinopathy candidiasis ectodermal dystrophy (APECED) is a rare autosomal recessive disease, caused by mutations of a single gene named autoimmune regulator gene (*AIRE*) which results in a failure of T cell tolerance within the thymus. Chronic mucocutaneous candidiasis, chronic hypoparathyroidism, and Addison's disease are the hallmarks of the syndrome. APECED is also characterized by several autoimmune endocrine and nonendocrine manifestations, and the phenotype is often complex. Moreover, even though APECED is a monogenic disease, its clinical picture is generally dominated by a wide heterogeneity both in the severity and in the number of components even among siblings with the same *AIRE* genotype. The variability of its clinical expression implies that diagnosis can be challenging, and a considerable delay often occurs between the appearance of symptoms and the diagnosis. Since a prompt diagnosis is essential to prevent severe complications, clinicians should be aware of all symptoms and signs of suspicion. The aim of this paper is to give an overview on the clinical presentation and diagnostic criteria of APECED and to focus on current knowledge on genotype-phenotype correlation.

## 1. Introduction

Autoimmune polyendocrinopathy candidiasis ectodermal dystrophy (APECED) is a rare autosomal recessive disease (OMIM 240300) with a complex clinical phenotype discovered over decades. APECED is the first multiple autoimmune disease that has been shown to be caused by mutations of a single gene named autoimmune regulator gene (*AIRE*), which maps to 21q22.3 [1] and encodes a 55-kDa protein that acts as a transcription regulator expressed in immune-related organs [2]. Immunologically, the disorder is characterized by lymphocytic infiltrate of target organs and appearance of serum autoantibodies against several defined tissue-restricted antigens, predicting or correlating with functional failure [3, 4]. The variability of its clinical expression implies that diagnosis can often be challenging. Since a prompt diagnosis is essential to prevent severe complications, clinicians should be aware of all symptoms and signs of suspicion. Aim of this paper is to give an overview on the clinical presentation and diagnostic criteria of APECED. Moreover, attention will be paid to the current knowledge on genotype-phenotype correlation. 

## 2. Clinical Presentation

APECED usually presents in childhood, but new disease components may appear even in the fifth decade of life. Clinical picture is generally characterized by a wide heterogeneity and the phenotype widely varies in the severity and in the number of components among affected subjects. This variability reflects the highly variable pattern of destructive autoimmune reactions toward different endocrine and non endocrine organs [5]. Chronic mucocutaneous candidiasis (CMC), chronic hypoparathyroidism (CH) and Addison's disease (AD), represent the clinical hallmark of the syndrome and the clinical diagnosis of APECED requires the presence of at least two of these three major components. CMC has been reported as the first sign to appear in most but not all series of reported patients. In fact, in a series of 23 Iranian Jewish patients only four had relatively mild oral, transient, candidiasis [6]. It is often followed by CH, before the age of 10 yrs, and later by adrenal insufficiency [2]. In addition to the classic triad (CMC, CH, AD), the phenotype of APECED includes several endocrine and nonendocrine autoimmune manifestations, which in a few cases may also precede the classical triad. In fact, 10 of 91 Finnish patients had one to three other components 0.1–14 years prior of the appearance of any of the major elements of the triad [7]. Within subjects with CMC, 21 patients had from one to six other components for 0.2–25 years before CH or AD appeared. CMC is a sign of the underlying immunodeficiency, thus being different in the pathogenesis from the multiple autoimmune manifestations of APECED. It preferentially affects the oral mucosa causing a mild form of intermittent angular keilitis. More severe cases include inflammation of most of the oral mucosa, hyperplastic CMC, and atrophic form with thin mucosa and leukoplakic areas. Oesophageal and intestinal candidiasis may also occur and is characterized by abdominal pain, flatulence, and diarrhea. Patients with long-lasting oral and/or esophageal candidiasis are at increased risk of esophageal squamous cell carcinoma [7]. In the Finnish series, 10.5% of patients over 25 years of age developed squamous cell carcinoma of the oral cavity or of the esophagus [7]. This indicates that the carcinoma is not rare in these patients and, therefore, candidiasis should be aggressively treated with topical antifungals together with good oral hygiene [8]. The critical mechanisms of the increased susceptibility to CMC in APECED patients are still poorly understood although autoantibodies to cytokines seem to be implicated in the pathogenesis. Recently, a role of specific neutralizing autoantibodies against the Th17-related cytokines IL-22 and IL-17F, and the concomitant loss of Th17 and Th22 cells, has been hypothesized in the pathogenesis of the CMC [9]. On the other hand, it should be mentioned that it has been hypothesized that chronic candida infection may trigger an autoimmune disease by inducing a chronic inflammation with the persistence of high levels of cytokines. 

CH is usually the first endocrine component. Symptoms of hypocalcemia may be vague with muscle cramps, mild paraesthesia, and clumsiness for long-time before the diagnosis is made and, sometimes, hypocalcemia may precipitate during febrile illness presenting with grand-mal type seizures. Candidate autoantigens previously linked to the development of hypoparathyroidism in APECED patients, as the calcium sensing receptor (Ca-S-R) has not been confirmed as relevant autoantigen [4, 10–13]. Recently, NALP5 (NATCH leucine-rich repeat protein 5) has been identified as the target for autoimmune attack in the parathyroid cells [14] in the context of APECED but autoantibodies against this antigen are exceptionally rare in isolated hypoparathyroidism [15]. 

AD appears most commonly between 5 and 15 years of age. Adrenal insufficiency may be asymptomatic for long-time as well as patients can report fatigue, weight loss, and increased pigmentation of mucous membrane and skin. When unrecognized adrenal crisis may represent a fatal event. Elevated plasma ACTH and/or renin together with low cortisol are the hallmarks of the disease. However, it should be highlighted that an increase in renin levels alone may be the first biochemical abnormality of AD, since the destructions of adrenal zona fascicualta and zona glomerulosa often are not simultaneous, the latter being the first layer targeted by the autoimmune attack [16]. The majority of patients with APECED may display autoantibodies against 21-hydroxilase even years before the clinical onset of the disease [4]. The detection of these antibodies calls for monitoring the adrenal function to prevent adrenal crisis.

The spectrum of endocrinopathies associated with APECED in addition to CH and AD includes hypergonadotropic hypogonadism, which is generally present only in affected females, type 1 diabetes, autoimmune thyroid diseases, and pituitary defects [17]. The occurrence of these manifestations is usually associated with a specific array of organ-specific autoantibodies that can appear quite before the overt clinical manifestation. 

The presence of ectodermal abnormalities is also common. The main ectodermal manifestations in APECED are dental enamel hypoplasia, pitted nail dystrophy, and alopecia. Keratopathy, vitiligo, calcifications of the tympanic membranes, and rash with fever can also be present [7, 17]. In young patients periodic maculopapular, morbilliform, or urticarial rash usually with fever can be part of the first manifestation and in most, appears before the age of 5 years [7]. Although the pathogenesis of ectodermal dystrophies seems to be autoimmune, no specific antibodies associated have been reported to date.

Ocular manifestations are keratopathy, dry eye, sublenticular cataract, iridocyclitis, retinal detachment and optic atrophy [18]. Among these, keratopathy can be a severe complication which, in the absence of appropriate treatment, may lead to blindness [7].

Furthermore, gastrointestinal autoimmunity in APECED may lead to autoimmune gastritis, autoimmune hepatitis, and intestinal disorders with chronic diarrhea alternating with obstipation [17]. Autoimmune hepatitis can vary from mild and self-limited to severe forms requiring treatment with immunosuppressants [7] and is characterized by the presence of peculiar immunological markers such as autoantibodies against cytochrome P450IA2 (CYP IA2), CYP2A6, and aromatic L-aminoacid decarboxylase (AADC) [19–21]. Malabsorption and steatorrhea can be the result of exocrine pancreatic failure [7]. Interestingly, the intestinal endocrine cells are also the targets of autoimmune attack, and, with this regard, it has been suggested that intestinal dysfunction can also be considered to be an endocrinopathy [22]. Gastrointestinal symptoms have been associated with the presence of autoantibodies against tryptophan hydroxylase (TPHAbs) [23]. Cholelitiasis can also occur [24]. 

Asplenia, tubulointerstitial nephritis, obstructive lung disease, vasculitis, Sjögren's syndrome, cutaneous vasculitis, hemolytic anemia, scleroderma, metaphyseal dysplasia, and celiac disease have also been reported to be associated with APECED [25, 26]. Autoantibodies directed against the potassium channel regulatory protein (KCNRG), found in epithelial cells of terminal bronchioles, have been suggested as a marker for pulmonary disease in APECED patients [27]. The autoimmune nature of renal destruction has been confirmed by examining biopsy samples and by determining antiproximal tubular autoantibodies [17, 28, 29]. Acquired asplenia, presenting in up to 20% of APECED patients [7], results in impaired immune responses to encapsulated bacteria and is a serious risk factor for developing septicemia [30]. Asplenia can be suspected on the basis of the presence of peripheral blood smears of Howell-Jolly cells. The pathogenesis of asplenia remains unknown.

Muscle disease is an additional component of APECED. Six cases have been so far described with very similar clinical features of progressive limb-girdle myopathy in the context of APECED [31]. Myopathy with axial muscle involvement may also lead to a respiratory involvement and, eventually, life-threatening respiratory failure [31].

To date, only 2 cases of encephalitis have been reported in the context of APECED one of them leading to a severe and life-threatening condition [32, 33].

Life expectancy of patients with APECED depends on the severity of the disease. The overall mortality of patients with APECED varies widely on the basis of the clinical spectrum. The most dangerous autoimmune manifestations are fulminant necrotizing hepatitis, severe malabsorption, and tubulointerstitial nephritis. Suboptimal hormonal substitution or inadequate management of addisonian crisis, may also increase the mortality risk. Furthermore, patients with long-lasting oral candidiasis are at increased risk of esophageal squamous cell carcinoma [7].

Disease targeted therapy is not currently available and the treatment mainly relies on hormone replacement and caring for clinical symptoms. So far, immunosuppressive therapy has only been used for potentially fatal disease such as hepatitis, nephritis, or severe malabsorption. New examples of immunomodulatory treatment of *Aire *knockout mouse both targeting T and B cells [34, 35] lend hope that such strategies could also be useful in the future for these patients. More recently, a monoclonal antibody against B cells, Rituximab, has been successfully used to treat pulmonary disease in APECED patients, raising the hope of applying it to all patients with APECED [36].

## 3. Diagnosis

The diagnosis of APECED is primarily based on the presence of two of the three most common clinical features: CMC, CH, and AD. The presence of only one component is sufficient for the diagnosis if a sibling is affected. However, the early clinical picture can be dominated by one of the minor components or the presence of only one major component and, in these circumstances, APECED can be misdiagnosed. Therefore, in children the presence of a minor component should prompt to carefully investigate for other symptoms. Candidiasis in childhood (and even more in adolescence or adulthood) is often underestimated as an initial symptom of a more complex disease. In a recent survey on 24 patients affected by APECED, Mazza et al. [32] underlined the considerable delay between the appearance of the first symptoms of APECED and the time of diagnosis of the disease with a mean diagnostic delay of about 10 years. 

The identification of causal genetic mutations in *AIRE* can confirm the diagnosis and may be helpful in those cases with atypical presentation. In about 95% of patients, two disease-causing *AIRE* mutations are detected [17]. Autoantibodies also constitute important diagnostic markers and may be in some cases predictive of a specific disease manifestation as detailed in Table 1 [3, 4, 13–15, 19–21, 24, 26–28, 37–43]. 

 Recently, neutralizing autoantibodies for type 1 interferons (INF) (IFN-*ω* and IFN-*α*) have been highly correlated with AIRE deficiency, regardless of the *AIRE* genotype, APECED features and duration. Therefore, they appear as a precious diagnostic tool to screen patients with unusual clinical manifestations of APECED instead of a more expensive and unjustified *AIRE* sequencing [44, 45]. In particular, Anti-IFN-*ω* antibodies seem to appear very early in life and their presence virtually confirms the diagnosis [45, 46]. Therefore, these autoantibodies have been recently included in the new diagnostic criteria for the diagnosis of APECED as reported by Husebye et al. [17] and shown in Table 2. 

## 4. Genetic Background

As already mentioned, APECED is caused by mutations in the transcriptional regulator, *AIRE*. AIRE is a crucial factor in the central tolerance for the right development of self-tolerance promoting clonal-deletion of self-reactive thymocytes. Within thymic medullary epithelial cells (mTECs), AIRE induces the expression of a broad repertoire of peripheral tissue antigens (PTAs) normally expressed in the periphery, eventually leading to the deletion of T-autoreactive cells [2, 47]. Therefore, the absence of AIRE results in impaired clonal deletion of self-reactive thymocytes, which attack a variety of organs. Moreover, there is now strong evidence for AIRE expression in peripheral tissues even if these levels are significantly lower than in thymic stromal cells. The lineage of extrathymic AIRE expressing cells have been described as both myeloid [48–52] and epithelial [53] in particular in lymph node, fetal liver, and appendix tissues. 

**Figure 1:**
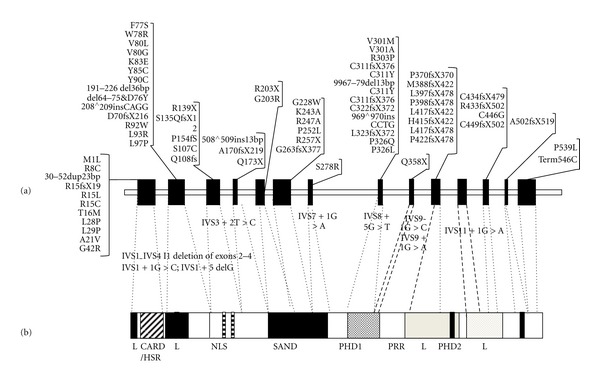
*AIRE* gene mutations (a) and functional domains of corresponding protein (b). Modified by Meloni et al [68].

Although APECED is rare, it is relatively more frequent in some populations (1 : 9000 in the Iranian Jews [6], 1 : 25000 in Finns [5, 54], and 1 : 14.400 in Sardinians [55]). It is also quite frequent in Norway (1 : 90.000) [56] and other regions of Italy [57]. Even though the most frequent mode of inheritance is autosomal recessive, an Italian family with APECED harbored a missense (G228W) mutation in the exon 6 in heterozygosity, indicating a dominant pattern of inheritance [58]. So far, over 70 different mutations of the *AIRE *gene have been documented in APECED patients [2] (Figure 1). Some different mutations have been found to be peculiar to certain populations. R257X is the most common mutation among Finnish and other European patients [59–61], 1094–1106 del113 (or 967–979 del13 bp) is the most common mutation in British [62], Irish [63], North America [64, 65], and Norwegian patients [56], and the Y85C is the only mutation found among Iranian Jews [66]. In Italy APECED shows an increased prevalence in various regions, in particular in Sardinia, Apulia, and Venetian area. Moreover, both in Sardinia and Apulia peculiar mutations of *AIRE *have been identified: the mutation R139X on exon 3 in Sardinia [21, 67] and the mutations W78R and Q358X on exon 2 and 9, respectively, in Apulia [68]. In the Veneto region, *AIRE* mutations (R257X and 979 del-13 bp on exon 6 and 8, respectively) were different from the other Italian regions but similar to that identified in Finnish and Anglo-saxon patients [69]. Among Sicilian patients, the typical mutation is R203X on exon 5 [70] but two novel mutations have been recently identified (S107C and Q108fs on exon 3) [71]. Although not showing a typical gene mutation, the patients from Campania exhibit a high frequency of mutations in the exon/intron 1 junction [57]. No *AIRE* gene mutation specific to Calabria has been found in patients with the disease [72]. 

## 5. Genotype-Phenotype Correlation

APECED is characterized by a wide variability of the clinical expression. In the largest reported series of 91 Finnish patients, a wide variation of the clinical phenotype and of the clinical course of APECED has been confirmed [7]. Later on, many other authors confirmed this phenotypic heterogeneity among several populations [54, 55, 57, 60, 63, 65, 69, 71, 73]. In the majority of these studies, no genotype-phenotype correlation has been found. However, several observations suggest that a genotype-phenotype correlation may exist. In fact, the Iranian Jewish patients' phenotype and genotype are peculiar of their population and differ markedly from others [6]. There is also evidence for a different sex prevalence of hypergonadotropic hypogonadism [7] and hypoparathyroidism [74]. Moreover, the G228W mutation has been associated with a peculiar phenotype. In fact, this mutation seems to elicit an unusually high risk of autoimmune thyroiditis (AT), while showing a lower penetrance for APECED [58].

Noteworthy, the clinical expression of the disease can be widely different even between siblings carrying the same mutation [75]. Such heterogeneity strongly suggests that disease-modifying genes, environmental factors, as well as immune system dynamics may play a role in modulating clinical expression of the syndrome. 

Recent studies revealed effects of additional genetic loci, in particular the human leukocyte antigen (HLA) complex on certain disease manifestations of APECED [76]. Associations with specific HLA haplotypes have been found for components like alopecia, AD, and type 1 diabetes in patients with APECED [76, 77]. These haplotypes are those associated with the common, non-APECED-related forms of that specific disorder. However, only a weak association has been observed between the HLA type and autoantibody specificities in APECED patients, suggesting that in APECED the HLA alleles do not have a strong influence on autoantibody formation *per se* [76]. 

Along with the central tolerance network, which is primarily involved in pathogenesis of APECED, several other peripheral mechanisms are capable of contributing to the control and regulation of the immune system [78]. These factors are involved in the maintenance of the homeostasis of peripheral tolerance of residual autoreactive clones, which escape negative selection within the thymus and play a significant role in preventing or minimizing reactivity to self-antigens. The peripheral tolerance recognizes, as possible mechanisms, the induction of functional anergy with inactivation of self-reactive T cells, deletion of autoreactive clones by apoptosis through interaction of Fas/FasL [79], and the suppressive action of regulatory T lymphocytes (Treg). An additional mechanism involved in controlling reactivity to self engages in the periphery is natural killer (NK) cells activity. Therefore, alterations dependant on one of the peripheral tolerance mechanisms [79] could contribute to the wide variability of APECED's clinical expression. To date, there are only few studies on the functionality of these immunological tolerance mechanisms in patients with APECED. A decrease of CD4+CD25+ Tregs in both adults and children with APECED has been reported [75, 80]. However, the reduction in circulating Tregs might also be secondary to the chronic fungal infection in these individuals and, therefore, their pathogenetic role in the disease still needs to be clarified [80]. Recently, several genetic, environmental, and molecular factors potentially implicated in the phenotypic variability of APECED have been investigated in two siblings affected with APECED. They were characterized by an extremely different phenotypic expression despite an identical *AIRE* (IVS1 + 1G > C; IVS1 + 5delG) [75]. In particular, the younger sister had a mild form of the syndrome while the older male developed a severe phenotype exhibiting an accelerated phase involving parathyroid, thyroid, oral mucosa, skin, liver, adrenal glands, bowel, and stomach culminating in a life-threatening posterior encephalopathy syndrome (PRES) [75]. PRES is a neurological condition characterized by acute encephalopathy with specific radiological findings, rarely reported in children [81]. The pathogenesis is still unclear, however there is evidence relating PRES with autoimmune diseases or use or use of immunosuppressants [57]. 

The exposure to infectious agents as rubella, Epstein Barr virus, cytomegalovirus, toxoplasma, varicella zoster virus, parvovirus B19, herpes simplex virus, and parainfluenza virus was ruled out as trigger factor [82]. Mechanisms of peripheral tolerance (Fas-induced apoptosis, number of TCD4+CD25+ regulatory cells, and natural killer activity) and HLA haplotype were compared in the two sibs and any significant difference was found. However, the chance is large that the two sibs differ in their genetic make-up and this could explain the difference in clinical course despite the same *AIRE* mutation. As for other Mendelian disorders, the interplay between multiple genetic, epigenetic, and environmental factors certainly play a role. 

## 6. Conclusions

APECED is a rare, complex, autoimmune disease. Diagnosis of APECED can be challenging and, although symptoms usually appear during the childhood, the diagnosis can be delayed up to the second decade of life. The reasons of difficulty in early recognition of these patients also rely on the heterogeneity of clinical spectrum which implies that genetic and environmental factors, other than AIRE, modulate the clinical expression. Moreover, it should be mentioned that the timing of the appearance of the individual disorders profoundly varies during the childhood, thus implying that most patients may develop new disease components in the 3rd–5th decade of life. A better understanding of these factors involved in the clinical expression of the disease could certainly improve current knowledge on the pathogenesis of APECED and help in identifying novel therapeutic targets. 

## Figures and Tables

**Table 1:** Main clinical features of APECED, specific related autoantibodies (when clearly associated with clinical manifestation) and usual period of life at onset of disease.

Clinical manifestation	Autoantibody specificities	Usual age at onset
Classic triad		
(i) Candidiasis	IL-17F, IL-22 [9]	Childhood
(ii) Hypoparathyroidism	NALP5, CaSR [13–15]	Childhood
(iii) Adrenal failure	P450c17, P450c21, P450scc [4, 37, 42, 83]	Childhood and adolescence
Other endocrine disorders		
(i) Ovarian failure	P450scc and P450c17 [4]	Adolescence to adulthood
(ii) Testicular failure	TSGA10 [84]	Adolescence to adulthood
(iii) Diabetes mellitus	IA-2, GAD65 [4, 42, 43]	Adulthood
(iv) Hypothyroidism	TG, TPO [38, 43]	Childhood to adulthood
(v) Hypopituitarism	TDRD6 [41]	Adolescence to adulthood
Ectodermal features		
(i) Alopecia	TH, hair follicles [4, 85]	Childhood to adulthood
(ii) Vitiligo	Melanocyte, SOX9, SOX10, AADC [39]	Childhood to adulthood
(iii) Keratopathy	Unknown	Childhood and adolescence
(iv) Enamel dysplasia	Unknown	Childhood
(v) Rash with fever	Unknown	Childhood
Gastrointestinal disorders		
(i) Gastritis/pernicious anemia	H^+^/K^+^ ATPase, IF [26, 43]	Childhood to adulthood
(ii) Severe obstipation	TPH, HDC [4, 23, 40]	Childhood to adulthood
(iii) Chronic diarrhea	TPH, HDC [4, 23, 40]	Childhood to adulthood
(iv) Immune Hepatitis	CYP1A2, CYP2AC, AADC, TPH, HDC [3, 4, 19–21]	Childhood
Lung manifestations	KCNRG [27]	Childhood to adulthood
Tubulointerstitial nephritis	Unknown	Childhood
Asplenia	Unknown	Childhood to adulthood

IL-17F: interleukin 17F, IL-22: interleukin 22, NALP5: NACHT leucine-rich-repeat protein 5, CaSR: calcium-sensing receptor, P450c17: Steroid 17-*α*-hydroxylase, P450c21: steroid 21-hydroxylase, P450scc: side-chain cleavage enzyme, TSGA10: testis-specific gene 10 protein, IA-2: islet antigen-2, GAD65: glutamic acid decarboxylase-65, Tg: thyroglobulin, TPO: thyroid peroxidase, TDRD6: tudor domain-containing protein 6, TH: tyrosine hydroxylase, AADC: aromatic l-amino acid decarboxylase, IF: intrinsic factor, TPH: tryptophan hydroxylase, HDC: histidine decarboxylase, CYP1A2: cytochrome P450 1A2, CYP2AC: cytochrome P450 2AC, KCNRG: potassium channel-regulating protein.

**Table 2:** New diagnostic criteria for the diagnosis of APECED as reported by Husebye et al. [17].

One of the following three criteria is necessary for a definitive diagnosis:
(i) presence of at least two of the three major components: chronic mucocutaneous candidiasis, hypoparathyroidism, or adrenal
Insufficiency
(ii) only one major component if a sibling is affected by APECED
(iii) disease-causing mutations in both *AIRE* genes
One of the following three criteria suggests a probable diagnosis:
(i) presence of one of the three major components (before 30 years of age) and at least one of the minor components
(ii) any component in the presence of anti-interferon antibodies
(iii) any component in the presence of antibodies against NALP5, AADC, TPH, or TH

*AIRE*: autoimmune regulator, NALP5: NACHT leucine-rich repeat protein 5, AADC: aromatic L-mino acid decarboxylase, TPH: tryptophan hydroxylase, TH: tyrosine hydroxylase.
